# Diagnostic potential of B‐cell translocation gene 2 in IgA nephropathy: Insights from molecular mechanisms

**DOI:** 10.1002/ccs3.70082

**Published:** 2026-07-20

**Authors:** Min Kou, Mo Dan, Juan Cheng, Sheng Ge, Ruimin Ren

**Affiliations:** ^1^ Department of Nephrology Shanxi Province Children's Hospital Taiyuan China; ^2^ The Third Clinical College of Shanxi University of Chinese Medicine Taiyuan China; ^3^ Department of Pediatrics Linfen People's Hospital Linfen China; ^4^ Department of Urology Third Hospital of Shanxi Medical University Shanxi Bethune Hospital Shanxi Academy of Medical Sciences Tongji Shanxi Hospital Taiyuan China

**Keywords:** B‐cell translocation gene 2, diagnostic biomarker, fibrosis, IgA nephropathy, immune microenvironment, machine learning

## Abstract

IgA nephropathy (IgAN) is a prevalent glomerular disease characterized by IgA deposition, chronic inflammation, and fibrosis, leading to end‐stage renal disease. This study explored B‐cell translocation gene 2 (BTG2) as a biomarker and treatment target for IgAN progression. Through integrated expression datasets and functional analyses, BTG2 emerged as a key gene with downregulation in renal tissues of IgAN patients, showing high diagnostic accuracy. BTG2 influenced the immune microenvironment by modulating T follicular helper cells and M0 macrophages. Moreover, BTG2 overexpression mitigated IgA deposition and fibrosis, whereas its silencing exacerbated these pathological changes. The study highlights BTG2 as a promising diagnostic tool and therapeutic target, shedding light on potential treatment strategies for IgAN.

## Graphical abstract

Diagnostic value of BTG2 and its immunoregulatory mechanism in IgAN. This study reveals that BTG2 improves immune microenvironment dysregulation, attenuates inflammation, and reduces fibrosis by promoting the expansion of T follicular helper cells, inducing macrophage polarization toward an M2 anti‐inflammatory phenotype, and inhibiting the TGF‐β1 pathway. We propose for the first time that BTG2 is a novel diagnostic target superior to existing markers, providing a new strategy for the precision diagnosis and treatment of IgAN.

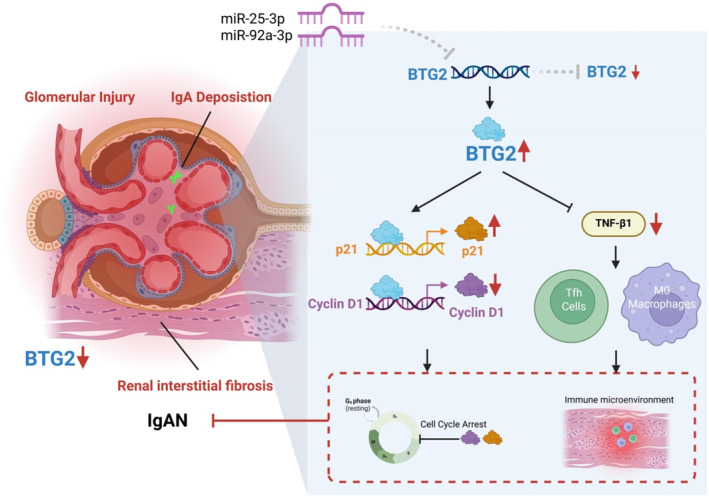

## 1 INTRODUCTION

IgA nephropathy (IgAN) is a common primary glomerular disease worldwide and an important cause of end‐stage renal disease, representing 20%–40% of renal biopsy cases.^1^, ^2^ The disease is primarily characterized by IgA deposition in the glomerular mesangial region, accompanied by mesangial cell proliferation, excessive extracellular matrix (ECM) accumulation, and chronic inflammation, ultimately progressing to glomerulosclerosis and interstitial fibrosis.^3^, ^4^ The pathogenesis of IgAN is complex and is thought to involve genetic susceptibility, mucosal immune dysregulation, complement system abnormalities, and oxidative stress.^5^ In particular, the formation of galactose‐deficient IgA1 (Gd‐IgA1) and its interaction with autoantibodies have been identified as key events in disease initiation.^6^, ^7^ Although the molecular mechanisms underlying IgAN have been extensively investigated, robust biomarkers and effective therapeutic targets remain lacking. Current clinical management largely relies on supportive therapies, including renin‐angiotensin system inhibitors and immunomodulatory agents, but their efficacy is limited.^8^ In recent years, gene expression profiling has offered new insights into the molecular pathogenesis of IgAN.^9^ Using high‐throughput sequencing and bioinformatics approaches, researchers have identified several differentially expressed genes (DEGs) associated with IgAN, including PLAU, FN1, and JUN; however, the functional validation of most candidate genes remains insufficient.^10^, ^11^ Therefore, there is an urgent need for in‐depth mechanistic studies to identify key regulatory factors involved in IgAN progression.

In the context of precision medicine for IgAN, identifying diagnostic biomarkers with high sensitivity and specificity is crucial. Currently used clinical markers for IgAN, such as proteinuria and serum creatinine, offer limited diagnostic value due to their lack of specificity and are insufficient for early disease assessment or prognosis prediction.^12^, ^13^ In recent years, researchers have employed gene expression profiling and machine learning algorithms to explore molecular biomarkers for IgAN; however, most studies have not validated their findings in independent datasets.^11^, ^14^ Moreover, existing research has predominantly focused on known inflammation‐ or nephropathy‐associated genes, often overlooking novel genes with antiproliferative or antifibrotic potential.^3^ B‐cell translocation gene 2 (BTG2), an antiproliferative factor, has been reported to inhibit tumor growth by regulating the cell cycle.^15^, ^16^ Although BTG2 has been shown to have protective roles in renal medullary hypertension and renal carcinoma,^17^, ^18^ its function in IgAN remains unclear. BTG2 is located on chromosome 1q32.1, and its promoter region contains multiple miRNA‐binding sites, including that of miR‐25‐3p, which has been reported to be significantly upregulated in the urine of patients with IgAN. These findings suggest that BTG2 may be involved in IgAN pathogenesis through miRNA‐mediated regulatory mechanisms.^19^, ^20^ Therefore, this study hypothesizes that BTG2 may act as a key regulatory factor in IgAN, with its dysregulated expression closely linked to disease progression, and holds promise as both a diagnostic biomarker and therapeutic target.

Uncontrolled cell proliferation and fibrosis are central pathological features of IgAN. As an antiproliferative gene, BTG2 has been reported to suppress cell cycle progression by upregulating p21 and downregulating cyclin D1, thereby inhibiting cellular proliferation.^21^, ^22^ In addition, BTG2 can antagonize the negative regulatory effects of certain miRNAs (e.g., miR‐92a‐3p), thereby blocking the TGF‐β1/Smad signaling pathway and suppressing the expression of fibrosis‐related genes such as α‐SMA and Collagen I/III.^23^, ^24^ In renal tissue, BTG2 may indirectly regulate renal fibrosis by shaping the immune microenvironment, particularly through effects on regulatory T cells and macrophages.^25^, ^26^ Notably, glomeruli in IgAN patients frequently exhibit increased infiltration of M1 and M2 macrophages,^27^ and BTG2 has been reported to interact with T follicular helper cells (Tfh cells), contributing to the maintenance of immune microenvironment homeostasis.^28^ Therefore, BTG2 may exert a protective role in IgAN through dual mechanisms: direct inhibition of fibrotic pathways and indirect regulation of immune responses. However, most existing evidence is derived from in vitro studies or animal models, and its clinical relevance remains to be validated. Moreover, limited studies have examined the association between BTG2 and IgAN‐specific pathological markers, such as IgA deposition and Gd‐IgA1, which constrains its potential clinical utility in disease diagnosis.

Current research on IgAN has primarily focused on characterizing the pathological phenotype and conducting single‐gene association analyses, with limited investigation into its molecular mechanisms.^29^, ^30^ Previous studies often relied on single datasets to identify DEGs, raising concerns about the stability and generalizability of potential biomarkers.^31^ Although machine learning algorithms such as least absolute shrinkage and selection operator (LASSO) regression and support vector machine recursive feature elimination (SVM‐RFE) have been applied for gene selection, their results were typically based on a single algorithm without cross‐validation using multiple methods, making them prone to overfitting.^14^ Furthermore, most studies have not validated the diagnostic performance of candidate genes in independent cohorts, thereby limiting their clinical translation potential. In terms of mechanistic research, current studies have predominantly focused on individual pathways, such as NF‐κB or Notch signaling, while overlooking the possible influence of pathway crosstalk on disease progression.^32^ For instance, BTG2 has been reported to inhibit cell proliferation and regulate fibrosis; however, its role in concurrently modulating these two key pathways remains unclear.^33^ It is also noteworthy that IgAN exhibits considerable pathological heterogeneity among patients, such as varying proportions of crescentic or sclerotic lesions, yet existing studies have not performed stratified analyses to assess BTG2 expression across different pathological subtypes—thereby weakening its clinical applicability.^34^ Therefore, a research strategy that integrates multi‐omics data, machine learning, and experimental validation is essential for identifying core regulatory factors in IgAN.

This study combined bioinformatic analysis with experimental validation to explore the regulatory role of BTG2 in IgAN and evaluate its clinical significance. Gene expression profiles related to IgAN were first obtained from the Gene Expression Omnibus (GEO) database, and key differentially expressed genes (DEGs) were identified using LASSO regression and SVM‐RFE. Their diagnostic performance was then validated in independent datasets. Subsequently, in vitro HK‐2 cell models and an in vivo mouse model of IgAN were employed to systematically evaluate the effects of BTG2 on cell proliferation (p21, cyclin D1) and fibrosis (TGF‐β1, α‐SMA, Collagen I/III). In addition, immunofluorescence and immunohistochemical analyses were conducted to determine the association between BTG2 and key pathological features of human IgAN, such as IgA deposition and mesangial proliferation, and to further investigate its potential involvement in immune microenvironment regulation. This study is expected to be the first to systematically elucidate the functional network of BTG2 in IgAN, thereby providing a novel theoretical basis for early diagnosis, disease monitoring, and targeted therapy. Moreover, by incorporating cross‐validation across multiple datasets and functional experimental analyses, the study significantly enhances the reliability and translational potential of its findings, with the prospect of advancing precision medicine for IgAN.

## 2 MATERIALS AND METHODS

### 2.1 Acquisition of datasets and identification of DEGs

Four IgAN‐related gene expression datasets, GSE115857, GSE16626, GSE93798, and GSE35487, were obtained from the GEO database. All datasets were derived from human renal tissue and generated using microarray platforms. GSE115857 and GSE16626 were integrated as the training cohort, whereas GSE93798 and GSE35487 were combined as the validation cohort. Batch effects were corrected with the ComBat function in the “SVA” package in R (v4.1.2). Differential expression analysis was conducted using the “limma” package, which includes core functions such as lmFit, eBayes, and topTable. Genes with *p <* 0.05 and |log fold change (FC)| > 1 were defined as DEGs. Volcano plots were generated with the “ggplot2” package to visualize the DEG results. Probe annotation and normalization of expression values were carried out according to the platform annotation files provided by GEO.

### 2.2 Functional enrichment of DEGs

Gene Ontology (GO) functional enrichment and Kyoto Encyclopedia of Genes and Genomes (KEGG) enrichment analyses were performed for the identified DEGs using the “clusterProfiler” package (v4.0.5), along with “enrichplot” (v1.14.2) and “ggplot2” for visualization. The functions enrichGO() and enrichKEGG() were applied with a significance threshold of *p*.adjust <0.05, using all detected genes as the background.

To assess the overall pathway activity differences between the IgAN and control groups, gene set enrichment analysis (GSEA) was performed using the GSEA() function in “clusterProfiler.” Genes were ranked according to log2FC, and the reference gene set c2.cp.kegg.v7.4.symbols.gmt was obtained from the MSigDB database. Pathways with FDR <0.25 and adjusted *p* < 0.05 were regarded as significantly enriched.

### 2.3 Identification and validation of key genes

To identify IgAN‐associated signature genes, two machine learning approaches, LASSO logistic regression and SVM‐RFE, were applied. LASSO analysis was performed using the “glmnet” package in R, whereas SVM‐RFE was conducted with the “e1071,” “kernlab,” and “caret” packages. Candidate gene sets obtained from both methods were intersected using a Venn diagram to determine the final set of key genes.

Subsequently, the classification performance of these key genes was evaluated in both the merged training and validation datasets. Receiver operating characteristic (ROC) curves were plotted using the “pROC” package, and the area under the curve (AUC) was calculated to assess diagnostic accuracy. All analyses were two‐tailed, and *p* < 0.05 was considered significant.

### 2.4 Immune cell profiling and correlation with key genes

To assess the characteristics of immune cell infiltration in IgAN tissues, we performed a quantitative analysis of 22 immune cell subtypes using the CIBERSORT algorithm (embedded script “CIBERSORT.R,” with 1000 permutations) based on gene expression matrices from the GEO datasets. The resulting immune cell fractions were further analyzed statistically. Differences in immune cell distributions between the IgAN and control groups were visualized using violin plots generated with the vioplot package, and correlations among immune cell subsets were displayed using the corrplot package.

To further investigate the association between key genes and immune infiltration, correlation analyses were performed between key gene expression and differentially abundant immune cell populations using the “ggpubr” and “ggExtra” packages. The results were presented as scatter plots, and Spearman correlation coefficients were calculated. A *p*‐value <0.05 was considered statistically significant.

### 2.5 Cell culture

In this study, HK‐2 and HMC cells were used as in vitro models to simulate the functional characteristics of renal tubular epithelial and mesangial cells under pathological conditions. Human kidney tubular epithelial cells (Homo sapiens; male; proximal tubule origin; official cell line name: HK‐2; RRID: CVCL_0302; HK‐2, obtained from ATCC, CRL‐2190, acquired in August 2024) and human mesangial cells (HMC; Homo sapiens; glomerular mesangial origin; official name: primary human mesangial cells; RRID: not available; HMC, FH0241, purchased from Fuheng Biology, Shanghai, acquired in August 2024) were employed. All cell cultures were routinely screened for *mycoplasma* contamination using a PCR‐based assay and were confirmed to be mycoplasma‐free prior to initiating experiments and throughout the experimental period (HK‐2 and HMC, all tests negative). HK‐2 cells were cultured in a DMEM/F12 medium (HyClone) supplemented with 10% fetal bovine serum (FBS, HyClone) at 37°C in a humidified atmosphere containing 5% CO_2_.^35^ HMC cells were maintained in the DMEM medium containing 10% FBS and were passaged twice before experiments to ensure uniform cell status.

### 2.6 Lentiviral transduction and selection of stable cell lines

Lentivirus‐mediated overexpression of BTG2 and knockdown of PPARα/FABP1 were employed to establish stably transfected cell models (Supporting Information S1: Figure S1A). All lentiviral constructs were synthesized and packaged by HanHeng Biotechnology Co., Ltd. (Shanghai, China) and carried a puromycin resistance gene for subsequent selection.

#### 2.6.1 BTG2 overexpression

HK‐2 cells were transduced with BTG2‐expressing lentivirus (LV‐BTG2, target sequence: 5′‐GACATGAGCCACGGGAAGGGAAC‐3′) or an empty vector control lentivirus (target sequence: 5′‐CAACAGCCACAACGTCTATAT‐3′). Cells were plated in 6‐well plates and transduced at 30%–50% confluence with an MOI of 20 and 8 μg/mL Polybrene. The medium was replaced after 6 h, followed by puromycin selection (2 μg/mL) 24 h later. Stable BTG2‐overexpressing cells were obtained after 72 h of selection (Supporting Information S1: Figure S1B,C).

#### 2.6.2 BTG2 knockdown

Three short hairpin RNA (shRNA) sequences targeting human BTG2 were designed and screened for knockdown efficiency. The sequences were as follows: shBTG2‐1: 5′‐CACTCACAGAGCACTACAAAC‐3′; shBTG2‐2: 5′‐GCAACTGACCTCTATGCAATA‐3′; shBTG2‐3: 5′‐CAGTGCTGTGACTTCAACATA‐3′; shNC (negative control [NC]): 5′‐TTCTCCGAACGTGTCACGT‐3′. HMC cells were seeded in 6‐well plates and infected at 30%–50% confluence with the corresponding lentiviral vectors at an MOI of 20 under the same conditions described above. Puromycin (2 μg/mL) was added after infection to select stably transduced cells. Total RNA was isolated after 5 days, and knockdown efficiency was assessed by quantitative real‐time PCR (RT‐qPCR) (Supporting Information S1: Figure S1D,E). The most effective sequences were used in subsequent experiments: shBTG2‐2 for HK‐2 cells and shBTG2‐1 for HMC cells. These were uniformly referred to as shBTG2 in all subsequent analyses.

### 2.7 Cell viability assay (CCK‐8 method)

HK‐2 and HMC cells were seeded in 96‐well plates (6 × 10^3^ cells/well). Cell viability was measured at 24, 48, and 72 h using the CCK‐8 assay. At each time point, 10 μL of CCK‐8 reagent (Beyotime, Shanghai) was added and incubated at 37°C for 2 h in the dark, followed by the measurement of absorbance at 450 nm. All experiments were performed in triplicate. Wells containing only culture medium and CCK‐8 reagent (without cells) served as blanks, and the final proliferation values were obtained by subtracting blank readings from sample measurements.

### 2.8 Preparation of Gd‐IgA1 (enzymatic deglycosylation of IgA1)

To obtain Gd‐IgA1, human serum‐derived IgA1 protein was subjected to enzymatic deglycosylation.

Specifically, human serum IgA1 protein (Sigma‐Aldrich, Cat# I4036) was first purified by dialysis against buffer. The purified protein was then incubated at 37°C for 16 h with β‐1,3‐galactosidase (1 U/mL, New England Biolabs, Cat# P0747S) and neuraminidase (1 U/mL, Sigma‐Aldrich, Cat# N2876). The reaction buffer contained 20 mM Tris‐HCl (pH 7.5), 50 mM NaCl, and 1 mM CaCl_2_.

After incubation, the reaction mixture was concentrated, and enzymes were removed using Amicon ultrafiltration tubes (10 kDa cutoff, Millipore). The final Gd‐IgA1 product was resuspended in sterile PBS for storage. The degree of galactose deficiency was confirmed by lectin‐based enzyme‐linked immunosorbent assay (ELISA) using LCA or HAA to detect GalNAc exposure, verifying successful deglycosylation before use in subsequent HMC stimulation experiments.

### 2.9 Lectin ELISA validation of Gd‐IgA1 glycosylation status

To verify the efficacy of enzymatic deglycosylation, Gd‐IgA1 was analyzed using a lectin‐based ELISA (Supporting Information S1: Figure S2A). Briefly, untreated IgA1 and enzyme‐treated Gd‐IgA1 were diluted to 2 μg/mL in carbonate buffer (pH 9.6) and coated onto high‐binding 96‐well ELISA plates, followed by overnight incubation at 4°C. After blocking with 1% BSA in PBS, horseradish peroxidase (HRP)‐conjugated Helix aspersa agglutinin (HAA, EY Laboratories) or Lens culinaris agglutinin (LCA, Vector Laboratories) was added at a final concentration of 1 μg/mL and incubated at 37°C for 1 h. Following thorough washing, the TMB substrate was added, and the absorbance was measured at 450 nm. The Gd‐IgA1 group exhibited higher OD_450_ values. As shown in Supporting Information S1: Figure S2B, Gd‐IgA1 yielded significantly increased OD_450_ readings with both HAA and LCA detection, indicating enhanced GalNAc exposure and confirming the successful generation of Gd‐IgA1.

### 2.10 Gd‐IgA1 stimulation for establishing an in vitro IgAN model

To establish an in vitro model of IgAN, human renal tubular epithelial cells (HK‐2) and HMC were cultured in serum‐free DMEM and stimulated with Gd‐IgA1 at concentrations of 1, 10, 100, or 1000 μg/mL for 24 h. Total RNA was then extracted to measure the mRNA expression of inflammatory cytokines, including TNF‐α, IL‐6, and MCP‐1, to determine the optimal induction concentration. The results showed that 100 μg/mL Gd‐IgA1 significantly upregulated the expression of inflammatory markers (Supporting Information S1: Figure S3A) while maintaining cell viability (Supporting Information S1: Figure S3B). Therefore, 100 μg/mL was selected as the standard treatment concentration for subsequent experiments.

Following the establishment of stably transfected cell lines, functional in vitro assays were performed in both HK‐2 and HMC cells. Eight experimental groups were established for each cell type: (1) empty vector control group (Vector); (2) empty vector + Gd‐IgA1 (Vector + Gd‐IgA1); (3) NC shRNA group (shNC); (4) shNC + Gd‐IgA1 (shNC + Gd‐IgA1); (5) BTG2 overexpression group (BTG2‐OE); (6) BTG2‐OE + Gd‐IgA1 (BTG2‐OE + Gd‐IgA1); (7) BTG2 knockdown group (shBTG2); and (8) shBTG2 + Gd‐IgA1 (shBTG2 + Gd‐IgA1). In all stimulation groups, cells were treated with 100 μg/mL Gd‐IgA1 for 24 h. Each experiment was performed with at least three biological replicates.

### 2.11 Animals and induction of IgAN

Male C57BL/6 mice (6–8 weeks old, SPF grade; purchased from GemPharmatech, ID: N000013) were housed in the SPF animal facility of our institution and acclimatized for two weeks. All animal procedures were approved by the Institutional Animal Care and Use Committee (Approval No. 2024082‐14).

#### 2.11.1 BTG2 overexpression intervention experiment

Mice were randomly assigned to four groups (*n* = 6/group): OV‐NC, IgAN, IgAN + OV‐NC, and IgAN + OV‐BTG2. The IgAN model was established according to a previously published protocol.^36^ Briefly, bovine serum albumin (BSA, 400 mg/kg; Sigma, USA) dissolved in saline was administered by oral gavage every other day for 8 weeks. Carbon tetrachloride (CCl_4_) mixed with castor oil was injected subcutaneously once weekly (0.15 mL per injection), with the proportion of CCl_4_ gradually increased over time. Lipopolysaccharide (LPS, 0.05 mg/mouse; Sigma, USA) was administered via tail vein injection during Weeks 6 and 8. Control mice received the same volume of normal saline. In Week 3, mice in the IgAN + OV‐NC and IgAN + OV‐BTG2 groups were injected via the tail vein with AAV9‐NC or AAV9‐BTG2, respectively (1 × 10^11^ vg/mouse in 200 μL). The remaining modeling procedures were identical among groups.

#### 2.11.2 BTG2 knockdown loss‐of‐function experiment

A separate set of mice was used to establish the BTG2 knockdown model, with the following groups: shNC, IgAN, IgAN + shNC, and IgAN + shBTG2. In the third week of modeling, mice in the intervention groups received tail vein injections of AAV9‐shBTG2 or AAV9‐NC (1 × 10^11^ vg/mouse). All viral vectors were synthesized and packaged by HanHeng Biotechnology (Shanghai, China).

In the 10th week after injection, mice were euthanized, and renal tissues were harvested. RT‐qPCR quantified BTG2 mRNA expression to confirm overexpression or knockdown efficiency (Supporting Information S1: Figure S4).

### 2.12 Flow cytometry

Single‐cell suspensions were isolated from renal tissues of mice in each IgAN group and filtered through 40 μm cell strainers before collection. Immune cell subsets were subsequently analyzed by flow cytometry.

Tfh cells were identified as CD4^+^/CXCR5^+^; macrophage subsets were defined as follows: M1 (F4/80^+^/CD86^+^) and M2 (F4/80^+^/CD206^+^). All antibodies were purchased from eBioscience, with catalog numbers and dilution ratios provided in Supporting Information S1: Table S1.

Flow cytometric analyses were performed using a BD LSRFortessa™. The optical stability of the instrument was verified daily using CS&T beads, and compensation matrices were established using UltraComp eBeads™ single‐stain controls, ensuring a coefficient of variation (CV) below 3%. Data were analyzed with FlowJo software version 10.8.

### 2.13 RNA extraction and RT‐qPCR

Total RNA was extracted from flow‐sorted cells using TRIzol® reagent (Invitrogen, USA). Briefly, cell pellets were resuspended in TRIzol (1:10, w/v) and lysed thoroughly. Chloroform (one‐fifth of the sample volume) was added, followed by vigorous shaking and a 10‐min incubation at room temperature. After centrifugation, the aqueous phase was collected for RNA precipitation with an equal volume of isopropanol. The RNA pellet was then washed twice with 75% ethanol, air‐dried, and dissolved in RNase‐free water.

RNA concentration and purity were measured using a NanoDrop™ 2000 spectrophotometer (Thermo Fisher Scientific), with an acceptable A260/A280 ratio >1.8. To eliminate genomic DNA contamination, 1 μg of RNA was treated with DNase I (Takara) at 37°C for 15 min. First‐strand cDNA was synthesized in a 20 μL reaction using PrimeScript™ RT Master Mix (Takara) at 42°C for 15 min, followed by enzyme inactivation at 95°C for 5 min.

Quantitative PCR (qPCR) was performed using TB Green® Premix Ex Taq™ II (Takara) on a QuantStudio™ 6 Flex Real‐Time PCR System (Applied Biosystems), with all reactions performed in triplicate. Primer sequences are provided in Supporting Information S1: Table S2. Amplification was conducted at 95°C for 30 s, followed by 40 cycles of 95°C for 5 s and 60°C for 30 s. Relative expression levels were normalized to β‐actin and quantified using the 2^−ΔΔCt^ method.

### 2.14 Dual‐luciferase reporter assay

Reporter plasmids containing the wild‐type BTG2 3′UTR with the predicted binding sites for miR‐25‐3p and miR‐92a‐3p were synthesized by GenScript (P/N: SC1016). Mutant reporter constructs were generated by introducing point mutations into the corresponding miRNA seed‐binding regions. Cells were seeded in 24‐well plates (5 × 10^4^ cells/well) and transfected using Lipofectamine™ 3000 (Invitrogen) with either wild‐type or mutant reporter plasmids (100 ng) along with a pRL‐TK plasmid (20 ng) for normalization and 50 nM of miR‐25‐3p mimic (RiboBio, Cat# miR0000125‐1) or miR‐92a‐3p mimic (RiboBio, Cat# miR0000126‐1). A mimic NC and an empty vector control group were included as NC. Luciferase activity was measured 48 h after transfection with the Promega Dual‐Luciferase Reporter Assay System, and relative activity was calculated as the firefly/Renilla ratio. All experiments were conducted in triplicate.

### 2.15 qPCR analysis of miRNA expression

Total RNA was extracted from mouse renal tissues, and miRNA reverse transcription was carried out using the miRCURY LNA RT Kit (Qiagen, Cat# 339340) in a 10‐μL reaction volume at 42°C for 60 min. Subsequently, miRNA expression levels were quantified using TaqMan probe‐based qPCR on a QuantStudio™ 6 Flex Real‐Time PCR System (Applied Biosystems), with TaqMan™ Universal Master Mix II (Thermo Fisher) as the reaction reagent. Probe IDs were as follows: miR‐25‐3p (002316), miR‐92a‐3p (000431), and U6 snRNA (internal control, probe ID 001973). All assays were conducted in triplicate, and expression levels were calculated using the 2^−ΔΔCt^ method.

### 2.16 Hematoxylin and eosin (H&E) staining and histopathological evaluation

Paraffin‐embedded tissue sections (4 μm thick) were deparaffinized and stained with H&E. Morphological evaluation was performed under a light microscope (Olympus BX60, Olympus) by experienced pathologists. For each mouse, 25 glomerular regions were randomly selected, and images were acquired and recorded at 200X magnification.

Renal pathological injury in IgAN model mice was evaluated using a quantitative morphological scoring system, including mesangial hyperplasia, basement membrane thickening, and tubulointerstitial changes. All histological assessments and image analyses were performed in a blinded manner. The evaluator was unaware of group allocation and treatment conditions to minimize bias.

Glomerular injury was semi‐quantitatively assessed according to mesangial proliferation and glomerulosclerosis using the following scale: 0, no obvious lesion; 1, involvement of <25% of glomeruli; 2, involvement of 25%–50% of glomeruli; and 3, involvement of >50% of glomeruli. For each mouse, 25 glomeruli were randomly selected and scored, and the mean score was calculated. All evaluations were performed independently by two blinded pathologists, and the average of their scores was used for analysis.

### 2.17 Periodic acid‐schiff (PAS) staining

Formalin‐fixed (10%), paraffin‐embedded renal tissues were sectioned at a thickness of 4 μm and stained with PAS reagent (Solarbio Co., Ltd., China). Stained sections were examined under an upright microscope (Carl Zeiss Meditec AG, Germany). Glomerular ECM was quantified by measuring PAS‐positive areas using ImagePro Premier 9 software. The mesangial matrix index was defined as the ratio of the PAS‐positive area to the total glomerular area.

### 2.18 Masson's trichrome staining

Formalin‐fixed (10%), paraffin‐embedded renal tissues were sectioned at a thickness of 4 μm according to the manufacturer's instructions. Following deparaffinization and rehydration, sections were first stained with hematoxylin to visualize nuclei. Subsequently, sections were stained with Ponceau S/acid fuchsin solution to colorize cytoplasm and muscle fibers red. A critical differentiation step using phosphomolybdic acid was performed, followed by counterstaining with aniline blue solution to specifically stain collagen fibers blue. Finally, sections were dehydrated, cleared, and mounted. Under a light microscope, blue deposits within the glomerular area were identified as collagen fibers, allowing for the assessment of the extent and distribution of collagen deposition.^37^

### 2.19 Immunofluorescence

Kidney tissues embedded in optimal cutting temperature (OCT) compound and stored at −80°C were sectioned at 8 μm using a cryostat. Frozen sections were blocked with PBS containing 5% goat serum and incubated with primary antibodies against IgA (1:500, Bioss, bs‐0774R) and Collagen I (1:100, Abcam, ab34710) overnight at 4°C. After washing, sections were incubated with secondary antibodies (1:200, Abcam, ab150081) for 1 h. Nuclei were counterstained with DAPI (1:1000, Thermo Fisher Scientific), and sections were mounted with antifade medium. Images were captured using a confocal laser scanning microscope (Nikon, Japan). Fluorescence intensity (mean gray value) was quantified using ImageJ 6.0 software (NIH, USA).

### 2.20 Western blot (WB) analysis

Kidney tissues harvested at the designated time points were homogenized in lysis buffer containing protease inhibitors (Roche, Cat# 4693159001 and 4906845001). Total protein concentration was determined using a BCA protein assay kit (Beyotime, China), and samples were adjusted to a final concentration of 5 mg/mL. Equal amounts of protein were separated by SDS‐PAGE and transferred onto PVDF membranes. After blocking with 5% nonfat milk in Tris‐buffered saline with 0.1% Tween‐20 (TBST), membranes were incubated overnight at 4°C with primary antibodies, followed by incubation with goat anti‐rabbit IgG H&L or goat anti‐mouse IgG H&L secondary antibodies (Zhongshan Golden Bridge, Beijing, China). Primary antibodies used were as follows: BTG2 (Cat# ab85051, Abcam, 1:1000) and β‐actin (Cat# ab8227, Abcam, 1:1000). Protein bands were detected using a Bio‐Rad ChemiDoc™ MP Imaging System, and band intensities were quantified with ImageJ 6.0 software (NIH, USA).

### 2.21 Statistical analysis

Data are presented as mean ± standard deviation (SD). Statistical significance was evaluated using Student's *t‐*test, one‐way ANOVA, Mann–Whitney *U* test, or Wilcoxon rank‐sum test, as appropriate. Spearman's rank correlation was used to evaluate associations between clinical features and bacterial taxa at the genus level. All statistical analyses were performed using SPSS version 26.0, with *p <* 0.05 (two‐tailed) considered statistically significant. For immunofluorescence quantification, six microscopic fields per group were analyzed. For WB, at least three kidney samples were included per group.

## 3 RESULTS

### 3.1 Differential gene expression and functional enrichment analyses in IgAN reveal aberrant immune regulation and inflammation‐associated pathways

To investigate the molecular mechanisms underlying IgAN, we analyzed gene expression profiles from the GSE115857 and GSE16626 datasets in the GEO database, annotated using the GPL14951 platform. These datasets comprised 107 IgAN samples and 14 healthy controls. DEGs were identified using predefined criteria, followed by functional enrichment analyses to examine their biological relevance and associated pathways.

**FIGURE 1:**
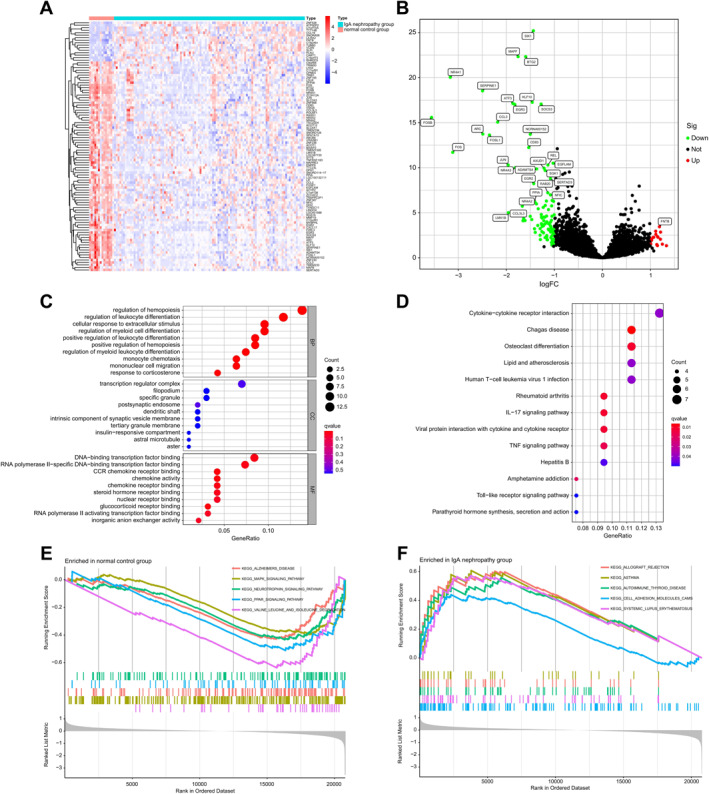
Identification and functional enrichment analysis of DEGs in IgAN. (A) Heatmap of DEGs between the IgAN group (*n* = 107) and the normal control group (*n* = 14), using the threshold |log_2_FC| > 1 and FDR <0.05. (B) Volcano plot of DEGs; red and blue dots indicate significantly upregulated and downregulated genes, respectively (*p <* 0.05). (C) GO enrichment analysis of DEGs, including BP, CC, and MF categories (FDR <0.05). (D) KEGG pathway enrichment analysis of DEGs (FDR <0.05). (E) GSEA of KEGG pathways in the normal control group (FDR <0.25). (F) GSEA of KEGG pathways in the IgAN group (FDR <0.25). (DEGs, differentially expressed genes; FC, fold change; FDR, false discovery rate; GO, Gene Ontology; GSEA, gene set enrichment analysis; KEGG, Kyoto Encyclopedia of Genes and Genomes).

A total of 120 DEGs were identified, including 16 significantly upregulated and 104 significantly downregulated genes. The distribution patterns of these DEGs were visualized through heatmaps and volcano plots (Figure 1A,B). GO analysis showed that these DEGs were mainly enriched in hematopoietic regulation, leukocyte differentiation, and myeloid cell differentiation in the biological process category, as well as transcription regulator complex and DNA‐binding transcription factor binding in the cellular component and molecular function categories, respectively (Figure 1C). KEGG analysis further indicated enrichment in cytokine–cytokine receptor interaction, Chagas disease, and osteoclast differentiation pathways (Figure 1D). Furthermore, GSEA suggested that IgAN pathogenesis may be closely associated with immune‐related pathways, including allograft rejection, asthma, autoimmune thyroid disease, cell adhesion molecules, and systemic lupus erythematosus (Figure 1E,F).

In summary, this study systematically analyzed the DEGs and their functional characteristics in IgAN, highlighting the critical roles of immune dysregulation and inflammation‐related pathways in the disease's development.

### 3.2 Machine learning‐based identification and multi‐dataset validation of diagnostic biomarkers for IgAN

To construct a robust molecular diagnostic model for IgAN, two machine learning methods, LASSO regression and SVM‐RFE, were used to identify candidate genes, followed by validation in independent datasets.

**FIGURE 2:**
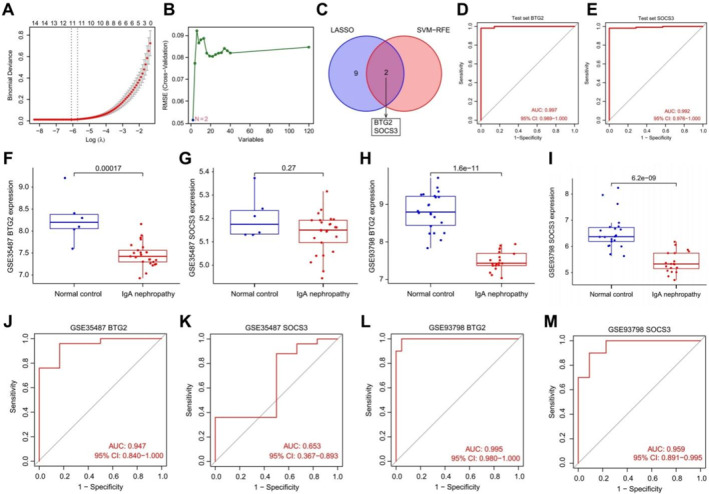
Identification and validation of diagnostic genes for IgAN using machine learning algorithms. (A) LASSO regression analysis for diagnostic gene selection (10‐fold cross‐validation used to determine the optimal λ value; 11 candidate genes highlighted in different colors). (B) Feature ranking by the SVM‐RFE algorithm (recursive feature elimination identified two key genes). (C) Venn diagram showing the overlap of genes identified by both algorithms, revealing BTG2 and SOCS3 as shared candidates. (D) ROC curve of BTG2 in the test dataset (AUC = 0.997). (E) ROC curve of SOCS3 in the test dataset (AUC = 0.992). (F) BTG2 expression in the GSE35487 dataset (IgAN group *n* = 25 vs. control group *n* = 6, *p <* 0.001). (G) SOCS3 expression in the GSE35487 dataset (IgAN group *n* = 25 vs. control group *n* = 6, *p* = 0.27). (H) BTG2 expression in the GSE93798 dataset (IgAN group *n* = 20 vs. control group *n* = 22, *p <* 0.001). (I) SOCS3 expression in the GSE93798 dataset (IgAN group *n* = 20 vs. control group *n* = 22, *p <* 0.001). (J) ROC analysis of BTG2 in the GSE35487 dataset (AUC = 0.947). (K) ROC analysis of SOCS3 in the GSE35487 dataset (AUC = 0.995). (L) ROC analysis of BTG2 in the GSE93798 dataset (AUC = 0.653). (M) ROC analysis of SOCS3 in the GSE93798 dataset (AUC = 0.959). (AUC, area under the curve; BTG2, B‐cell translocation gene 2; IgAN, IgA nephropathy; LASSO, least absolute shrinkage and selection operator; ROC, receiver operating characteristic; SOCS3, suppressor of cytokine signaling 3; SVM‐RFE, support vector machine‐recursive feature elimination).

LASSO analysis yielded 11 candidate diagnostic genes (Figure 2A), whereas SVM‐RFE selected 2 key genes (Figure 2B). Specifically, genes identified by LASSO regression but not retained by SVM‐RFE included SIK1, KLF10, EGR3, FOSB, CCL3, REL, RAB20, TMEM194, and TSC22D4. Among these, CCL3 and REL are associated with inflammatory responses; KLF10, EGR3, FOSB, and TSC22D4 are primarily involved in transcriptional regulation and cellular stress processes; SIK1 and RAB20 are related to metabolic regulation or vesicle transport, whereas the function of TMEM194 remains unclear. These genes exhibited inconsistent expression trends and fluctuating diagnostic efficacy across different validation datasets, leading to their exclusion during the SVM‐RFE feature refinement process. BTG2 and suppressor of cytokine signaling 3 (SOCS3) were ultimately pinpointed as the most promising diagnostic biomarkers through cross‐validation between the two algorithms (Figure 2C). In the test set, both genes demonstrated excellent diagnostic performance (BTG2 AUC = 0.997; SOCS3 AUC = 0.992) (Figure 2D,E). Further cross‐platform validation revealed that BTG2 expression was significantly downregulated in the IgAN group in both the GSE93798 (20 IgAN vs. 22 control) and GSE35487 (25 IgAN vs. 6 control) datasets (both *p <* 0.001) (Figure 2F,H), with consistently high diagnostic accuracy (AUC = 0.947 and 0.995, respectively) (Figure 2J,L). In contrast, SOCS3 expression showed dataset‐dependent variability (*p* = 0.27 in GSE35487 vs. *p <* 0.001 in GSE93798) (Figure 2G,I), and its diagnostic performance fluctuated markedly across datasets (AUC range: 0.653–0.959) (Figure 2K,M).

By integrating machine learning with validation in multicenter datasets, this study systematically demonstrated that BTG2 is consistently downregulated in IgAN and shows strong diagnostic performance, outperforming SOCS3 as a diagnostic biomarker.

### 3.3 BTG2 expression is downregulated in in vivo and in vitro models of IgAN

**FIGURE 3:**
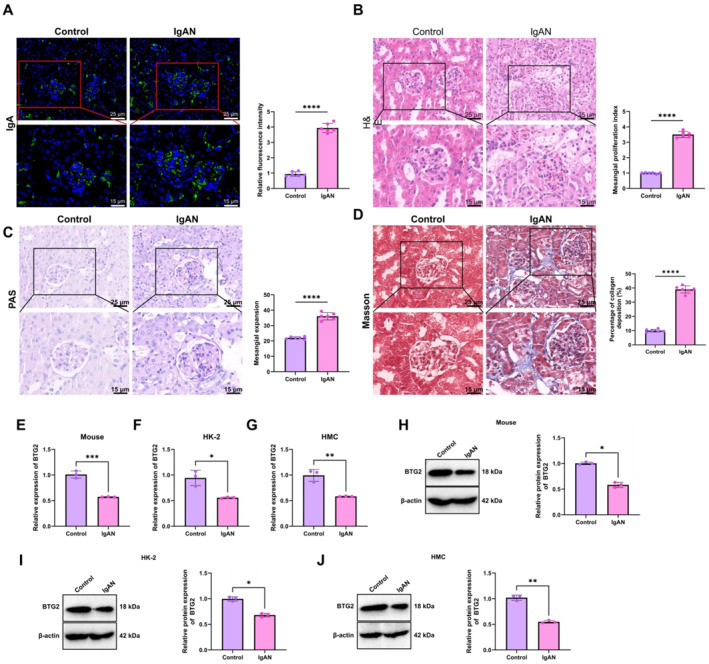
Expression analysis of BTG2 in in vivo and in vitro models of IgAN. (A) Immunofluorescence staining of renal tissues from IgAN mice showing IgA deposition (green) and DAPI‐labeled nuclei (blue); *n* = 6 per group; scale bar = 25 μm (upper) and 15 μm (lower). Right panel: quantification of fluorescence intensity. (B) H&E staining of mouse renal tissues evaluating glomerular and tubular pathological changes; scale bar = 20 μm (upper) and 15 μm (lower); *n* = 6. The right panel shows the quantification of mesangial expansion scores. (C) PAS staining of mouse renal tissues assessing glomerular mesangial proliferation and matrix expansion; scale bar = 25 μm (upper) and 15 μm (lower); *n* = 6. The right panel shows the quantification of mesangial expansion scores. (D) Masson's trichrome staining assessing collagen deposition within the glomeruli; scale bar = 25 μm (upper) and 15 μm (lower); *n* = 6. (E–G) Quantitative PCR analysis of BTG2 mRNA expression in renal tissues of IgAN mice, and in HK‐2 and HMC cells stimulated with Gd‐IgA1; *n* = 3. (H–J) Western blot analysis of BTG2 protein expression in IgAN mouse renal tissues, HK‐2 cells, and HMC cells; β‐actin used as loading control; *n* = 3. Statistical analysis was performed using one‐way ANOVA followed by Tukey's post hoc test. Data are presented as mean ± SD. *p < 0.05, **p < 0.01, ***p < 0.001, ****p < 0.0001. BTG2, B‐cell translocation gene 2.

To investigate the expression pattern of BTG2 in IgAN, we first established a murine model of IgAN and an in vitro Gd‐IgA1 stimulation model. Immunofluorescence and PAS staining confirmed increased glomerular IgA deposition and significant mesangial proliferation in the model groups, indicating successful model construction (Figure 3A–C). Masson's trichrome staining showed marked collagen deposition in the glomeruli of IgAN model mice (Figure 3D). qPCR and WB analyses further revealed that BTG2 mRNA and protein expression were significantly decreased in renal tissues from IgAN mice and in Gd‐IgA1‐treated HK‐2 cells compared with controls (Figure 3E–J), suggesting that BTG2 may play a critical role in IgAN pathogenesis.

### 3.4 BTG2 exerts a protective role in IgAN by modulating immune responses and suppressing inflammatory cytokines

**FIGURE 4:**
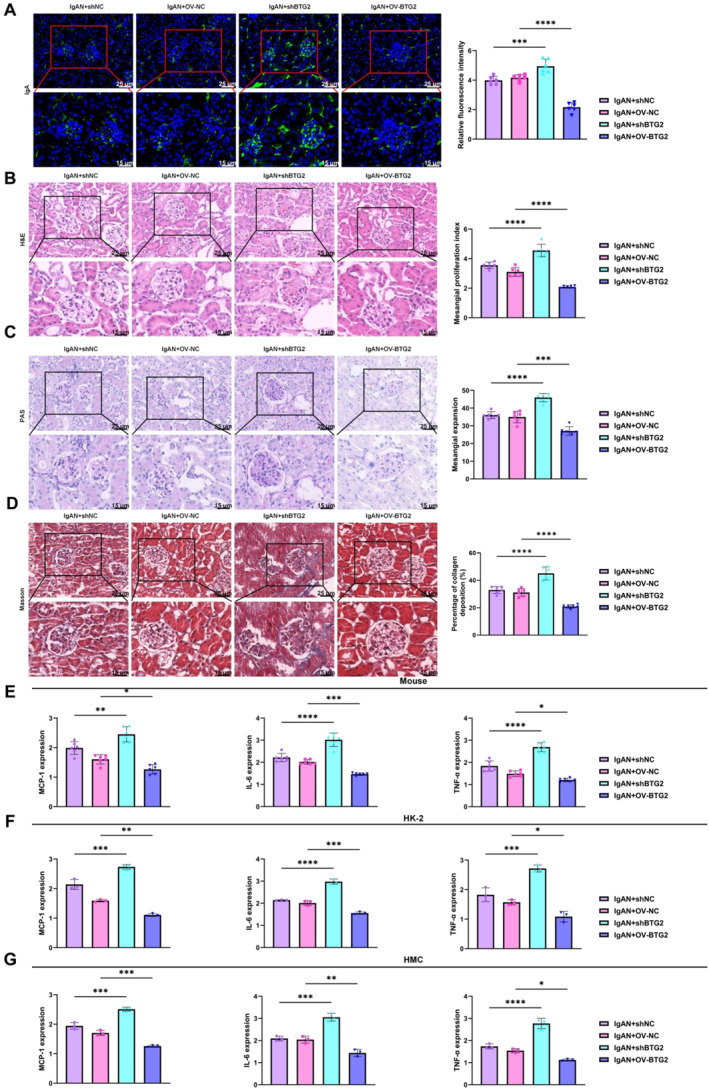
Pathological impact of BTG2 on IgAN mouse and cell models. (A) IgA immunofluorescence staining: Detection of glomerular IgA deposition in mouse renal tissues across different experimental groups using immunofluorescence; *n* = 6 per group. (B) PAS staining: Evaluation of mesangial hyperplasia in mouse renal tissues via PAS staining; *n* = 6 per group. (C) H&E staining: Assessment of inflammatory cell infiltration and tissue injury in mouse renal tissues using H&E staining. The extent of infiltration was quantified using image analysis; *n* = 6 per group. (D) Masson's trichrome staining: Assessment of collagen deposition within glomeruli; scale bar = 25 μm; *n* = 6 per group. (E) Relative mRNA expression levels of TNF‐α, IL‐6, and MCP‐1 in renal tissues from IgAN mice under different treatment conditions; *n* = 6. (F) Relative mRNA expression of TNF‐α, IL‐6, and MCP‐1 in HK‐2 cells across treatment groups; *n* = 3. (G) Relative mRNA expression of TNF‐α, IL‐6, and MCP‐1 in HMC cells across treatment groups; *n* = 3. Statistical analysis was performed using one‐way ANOVA with Tukey's post hoc test. Significance levels: *p < 0.05, **p < 0.01, ***p < 0.001, and ****p < 0.0001.

Following the confirmation of successful model establishment and downregulation of BTG2, we further explored the immunomodulatory function of BTG2 in IgAN progression. WB and qPCR analyses of mouse glomerular tissues revealed that BTG2 protein and mRNA expression were significantly reduced in the IgAN + shBTG2 group compared to the IgAN + shNC group. Conversely, BTG2 protein and mRNA levels were significantly increased in the IgAN + OV‐BTG2 group compared to the IgAN + OV‐NC group (Supporting Information S1: Figure S5A,B). Immunofluorescence staining for IgA revealed a marked increase in glomerular IgA deposition in IgAN model mice. However, BTG2 overexpression significantly reduced IgA deposition compared to the IgAN group, whereas BTG2 silencing exacerbated it. These findings indicate that BTG2 overexpression effectively alleviates IgA deposition, whereas its silencing aggravates this process (Figure 4A).

PAS staining further showed pronounced mesangial proliferation in IgAN model mice, which was notably attenuated in the BTG2 overexpression group. In contrast, BTG2 silencing failed to mitigate mesangial expansion, supporting a protective role of BTG2 in glomerular injury (Figure 4C). H&E staining corroborated these findings, demonstrating reduced renal inflammation and tissue damage in the BTG2 overexpression group, whereas BTG2 silencing resulted in more severe inflammatory cell infiltration and structural damage. These results indicate that BTG2‐mediated immunoregulation may suppress IgAN progression (Figure 4B). Masson's trichrome staining further showed that BTG2 overexpression reduced glomerular collagen deposition and renal fibrosis, whereas BTG2 knockdown aggravated these pathological changes (Figure 4D).

qPCR analysis showed that the mRNA levels of TNF‐α, IL‐6, and MCP‐1 were significantly increased in the IgAN group. These inflammatory cytokines were markedly reduced by BTG2 overexpression but further elevated after BTG2 knockdown (Figure 4E). In addition, WB and qPCR analyses in HK‐2 and HMC cells confirmed that BTG2 expression at both the protein and mRNA levels was significantly lower in the IgAN + shBTG2 group than in the IgAN + shNC group, whereas it was significantly higher in the IgAN + OV‐BTG2 group than in the IgAN + OV‐NC group (Supporting Information S1: Figure S5C–F). Consistent with these findings, in vitro experiments further demonstrated that BTG2 overexpression significantly suppressed TNF‐α, IL‐6, and MCP‐1 expression, whereas BTG2 silencing enhanced the expression of these inflammatory markers in both HK‐2 and HMC cells (Figure 4F,G). Collectively, these results suggest that BTG2 exerts an important immunoregulatory effect in IgAN by attenuating inflammatory responses and modulating the immune microenvironment.

### 3.5 Immune microenvironment characteristics of IgAN and the immunomodulatory role and molecular mechanism of BTG2

Immune cell infiltration is closely involved in the pathogenesis of IgAN, but changes in specific immune cell subsets and their associations with key regulatory genes remain unclear. This study systematically analyzed differences in immune cell composition between IgAN patients and healthy controls with a particular focus on the potential immunomodulatory role of the candidate diagnostic gene BTG2.

**FIGURE 5:**
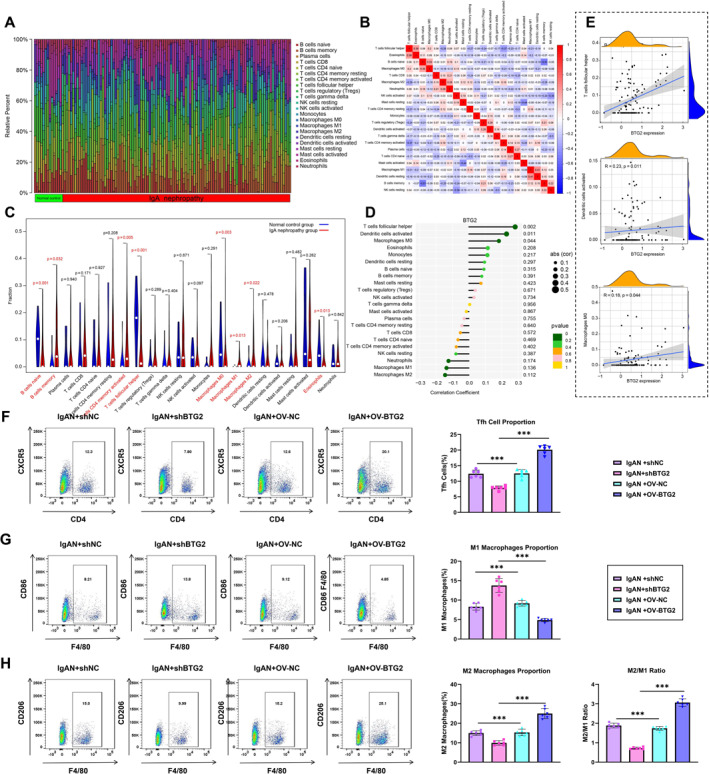
Immune cell infiltration patterns and BTG2 gene analysis in IgAN. (A) Proportional analysis of 22 immune cell types using the CIBERSORT algorithm (IgAN group *n* = 107 vs. control group *n* = 14). (B) Pearson correlation heatmap of immune cell interactions (|r| > 0.3, *p <* 0.05). (C) Violin plots comparing infiltration levels of 22 immune cell subsets using the Wilcoxon rank‐sum test. (D) Spearman correlation network between BTG2 expression and immune cell infiltration (node size represents the magnitude of |r|). (E) Spearman correlation scatter plots showing associations between BTG2 expression and Tfh cells, activated dendritic cells, and M0 macrophages (r and *p* values indicated). (F) Flow cytometry analysis showed that BTG2 knockdown decreased the proportion of Tfh cells compared with the IgAN + shNC group, whereas BTG2 overexpression increased the proportion of Tfh cells compared with the IgAN + OV‐NC group; *n* = 6 per group. (G–H) Flow cytometry analysis showed that BTG2 knockdown increased M1 macrophages and decreased M2 macrophages and the M2/M1 ratio compared with the IgAN + shNC group, whereas BTG2 overexpression decreased M1 macrophages and increased M2 macrophages and the M2/M1 ratio compared with the IgAN + OV‐NC group; *n* = 6 per group. Statistical analysis was performed using one‐way ANOVA with Tukey's post hoc test. Significance levels: **p < 0.01 and ***p < 0.001. BTG2, B‐cell translocation gene 2.

Immune infiltration profiling revealed marked differences in the immune microenvironment between the IgAN and control groups (Figure 5A). Specifically, the IgAN group exhibited increased infiltration of memory B cells, activated memory CD4^+^ T cells, and M1/M2 macrophages, whereas naïve B cells and Tfh cells were reduced (Figure 5C). Correlation analysis showed that BTG2 expression was positively associated with Tfh cells (*r* = 0.28, *p* = 0.0017), activated dendritic cells (*r* = 0.23, *p* = 0.011), and M0 macrophages (*r* = 0.18, *p* = 0.044) (Figure 5D,E). Moreover, the interaction network of 22 immune cell types revealed a significant positive correlation cluster involving naïve B cells and M0 macrophages (Figure 5B).

Flow cytometry further supported the immunoregulatory role of BTG2 in IgAN. In BTG2‐overexpressing IgAN mice, the proportion of Tfh cells was significantly higher than that in the IgAN group. Additionally, the percentage of M2 macrophages was markedly elevated, whereas M1 macrophage levels were relatively reduced, resulting in a significantly higher M2/M1 ratio (Figure 5F–H). These findings indicate that BTG2 overexpression promoted the expansion of Tfh cells and polarization toward the M2 macrophage phenotype, thereby exerting a protective effect in IgAN‐related immune responses.

Conversely, BTG2‐deficient mice exhibited pronounced immune dysregulation, characterized by a substantial decrease in Tfh cells, increased infiltration of M1 macrophages, and reduced levels of M2 macrophages, resulting in a markedly decreased M2/M1 ratio. These observations suggest that BTG2 depletion suppresses Tfh cell expansion, enhances M1 macrophage activation, and impairs M2 polarization, thereby exacerbating immune imbalance and the pathological progression of IgAN.

Collectively, these findings suggest that BTG2 may exert a protective effect in IgAN by regulating the immune microenvironment, particularly by promoting Tfh cell expansion and M2 macrophage polarization. In contrast, BTG2 knockout contributed to disease progression by impairing these immune responses.

### 3.6 Dual regulatory role of BTG2 in IgAN immune responses via modulation of miR‐25‐3p and miR‐92a‐3p mediated immune microenvironment Remodeling

**FIGURE 6:**
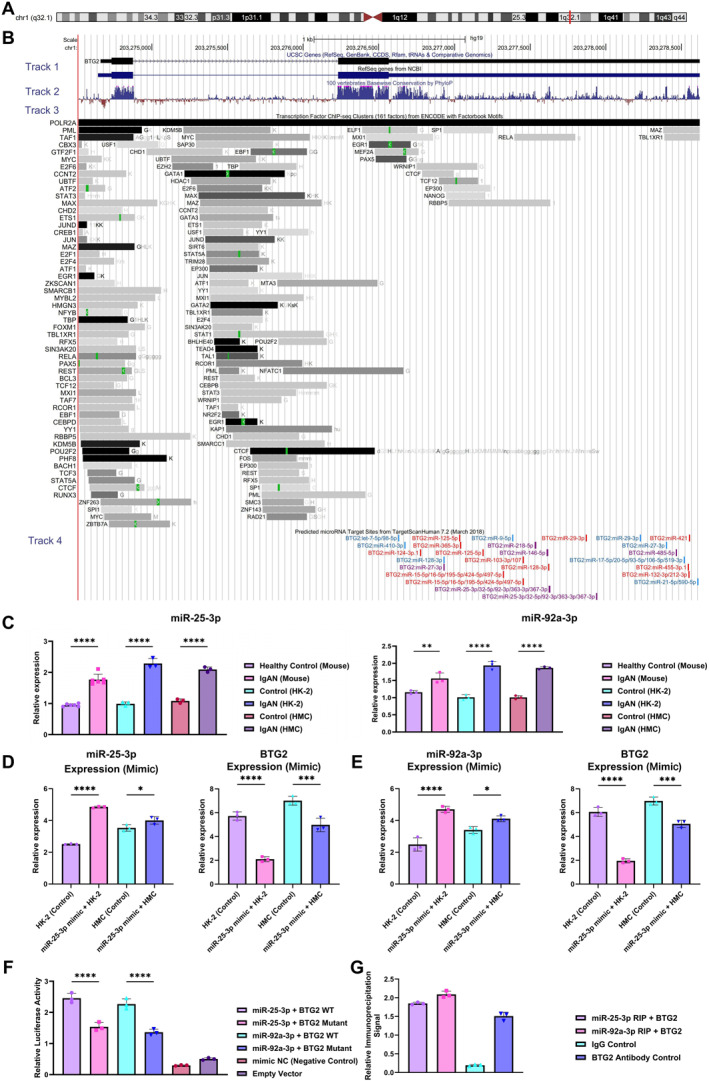
Genomic localization and functional analysis of the BTG2 gene. (A) Genomic localization of BTG2 (UCSC Genome Browser GRCh37/hg19, chr1:203, 274, 664–203, 278, 729). (B) Genomic feature analysis of BTG2: (1) exon structure; (2) PhyloP conservation scores across 100 vertebrate species; (3) ENCODE transcription factor ChIP‐seq clustering; (4) predicted miRNA binding sites from TargetScanHuman 7.2. (C) Expression changes of miR‐25‐3p and miR‐92a‐3p in vivo and in vitro IgAN models (in vitro *n* = 6, in vivo *n* = 3). (D) Relative expression of miR‐25‐3p and BTG2 after transfection with miR‐25‐3p mimic/agonist in HK‐2 and HMC cells (*n* = 3). (E) Relative expression of miR‐92a‐3p and BTG2 after transfection with miR‐92a‐3p mimic/agonist in HK‐2 and HMC cells (*n* = 3). (F) Dual‐luciferase reporter assay confirming direct interactions between miR‐25‐3p, miR‐92a‐3p and BTG2 (*n* = 3). (G) RIP assay validating the direct binding of miR‐25‐3p and miR‐92a‐3p to BTG2 (*n* = 3). BTG2, B‐cell translocation gene 2.

Molecular analysis revealed that the BTG2 gene, located on chromosome 1q32.1, is highly conserved (Figure 6A). As an antiproliferative factor, BTG2 inhibits tumor progression by regulating cell cycle and apoptosis. In the kidney, it may counteract the negative regulatory effects of microRNAs such as miR‐25‐3p (upregulated in IgAN) and miR‐92a‐3p (Figure 6B), thereby mitigating inflammation and interstitial hyperplasia‐mediated renal injury. Previous studies have demonstrated BTG2's protective role in renal medullary hypertension and renal carcinoma^38^; this study, for the first time, extends its functional relevance to IgAN, suggesting its therapeutic potential through the dual modulation of immune microenvironment reprogramming and pathological cell proliferation.

Further experiments confirmed that miR‐25‐3p and miR‐92a‐3p were significantly upregulated in both the IgAN mouse model and the in vitro IgAN models established in HK‐2 and HMC cells (Figure 6C). This upregulation likely downregulates BTG2 expression, thereby affecting cellular proliferation, apoptosis, and immune cell infiltration within the immune microenvironment.

To further validate this mechanism, we transfected HK‐2 and HMC cells with miR‐25‐3p and miR‐92a‐3p mimics. Overexpression of miR‐25‐3p and miR‐92a‐3p via mimics/agonists increased miRNA expression and decreased BTG2 expression in HK‐2 and HMC cells (Figure 6D,E). These results indicate that miR‐25‐3p and miR‐92a‐3p may suppress BTG2 expression, thereby influencing the renal immune microenvironment and modulating immune responses and pathological progression in IgAN.

Moreover, dual‐luciferase reporter assays and RNA immunoprecipitation (RIP) further confirmed the direct interaction between miR‐25‐3p, miR‐92a‐3p, and BTG2 (Figure 6F–G). Both miRNAs directly bound to the BTG2 3′UTR, supporting their inhibitory effect on BTG2 expression and providing mechanistic insights into their regulation of the immune microenvironment.

In summary, miR‐25‐3p and miR‐92a‐3p negatively regulate BTG2 expression, thereby modulating the renal immune microenvironment and influencing immune responses and disease pathology in IgAN.

## 4 DISCUSSION

The pathogenesis of IgAN is largely driven by the excessive production of Gd‐IgA1, which forms immune complexes with autoantibodies and deposits in the glomerular mesangium. This process promotes mesangial proliferation, complement activation, and inflammatory mediator release, ultimately contributing to disease onset.^39^, ^40^, ^41^, ^42^ Therefore, the present study sought to identify specific diagnostic genes and immune cell infiltration characteristics in IgAN, thereby providing a theoretical foundation for elucidating its pathogenesis, diagnosis, and therapeutic strategies.

Park et al. reported that spleen tyrosine kinase may play a pathogenic role in IgAN.^43^ Liu et al. identified 10 key genes—SYN1, SYT4, RBFOX1, KCNC1, VAMP2, FBXO11, ASB9, SYT9, KLHL5, and KRAS—in peripheral blood mononuclear cells from IgAN patients, which were closely associated with inflammation‐related glomerular transcriptomic pathways.^44^, ^45^ Jiang et al. found that PLAU, JUN, and FOS were linked to neutrophil function and IgAN development.^46^ Noor et al. reported that nine genes (FN1, EGR1, FOS, JUN, SERPINE1, MMP2, ATF3, MYC, and IL1B) and hsa‐miR‐144‐3p were associated with the diagnosis and treatment of IgAN.^47^ Qian et al. reported multiple genes related to IgAN prognosis, including JUN/JUNB, FOS, NR4A1/2, EGR1, and FOSL1/2.^48^ Notably, the genes JUN and FOS have been consistently reported in three independent studies.^46^, ^47^, ^48^ However, these genes have not yet been analyzed using ROC curves or validated across independent datasets, which limits their applicability. In our study, analysis based on both LASSO and SVM‐RFE algorithms identified BTG2 and SOCS3 as hub or key genes in IgAN. Compared with SOCS3 (AUC = 0.653 in GSE35487), BTG2 demonstrated greater diagnostic value (AUC = 0.947 in GSE35487 and AUC = 0.995 in GSE93798). Therefore, we selected BTG2 as the primary diagnostic gene for IgAN.

BTG2 is an antiproliferative gene located on chromosome 1q32.1 and contains two exons (Figure 6A). It is highly conserved across species (Figure 6B)^49^ and is known to regulate cell cycle progression, apoptosis, and differentiation. Additionally, BTG2 has been shown to suppress both lymphoid malignancies and solid tumors.^22^ Its function is also implicated in renal medullary hypertension,^18^ and it acts as a negative regulator of cardiomyocyte hypertrophy and adipogenesis.^50^, ^51^ The downregulation of BTG2 has been linked to renal cancer progression, whereas its overexpression inhibits renal cancer cell growth, migration, and invasion in vitro.^52^, ^53^ These findings suggest that the growth inhibition of anti‐IgM‐induced murine B lymphoma cells may be mediated by the upregulation of BTG2.^54^ Restoring BTG2 expression also helps suppress proliferation in BTG2‐deficient breast and prostate cancer cells.^55^, ^56^ To date, the biological role of BTG2 in IgAN has not been previously reported.

Transcription factors and microRNAs regulate the expression and function of BTG2 (Figure 6A). Notably, miR‐25‐3p is significantly upregulated in the urine of patients with IgAN.^57^ Both miR‐92a‐3p and miR‐15a have been reported to promote breast cancer cell proliferation and metastasis or enhance renal cell carcinoma progression by downregulating BTG2.^23^, ^58^ Inflammation and mesenchymal cell proliferation may contribute to renal injury.^59^, ^60^ In this study, we confirmed that miR‐25‐3p and miR‐92a‐3p directly target the 3′UTR of BTG2, and that BTG2 may function as a molecular “sponge” for these miRNAs. Notably, miR‐25‐3p is known to target STAT3, a key regulator of Tfh cell differentiation, suggesting that BTG2 overexpression may indirectly enhance STAT3 signaling by antagonizing this miRNA, potentially explaining the observed increase in Tfh cell proportions.^61^ The identification of this regulatory axis provides a novel target for miRNA‐based therapeutic strategies in IgAN. Thus, BTG2, as an antiproliferative factor, may help mitigate renal injury.

The production of IgA1 is regulated by cytokines secreted by B cells, T cells, dendritic cells, and monocytes.^62^ Our study demonstrated an increased number of both M1 and M2 macrophages in patients with IgAN, consistent with findings reported by Wei et al.^63^ Zeng et al. reported a marked decrease in both the number and cytotoxic activity of NK cells in peripheral blood mononuclear cells from patients with IgAN.^64^ In contrast, our analysis showed no significant difference in NK cell abundance between the IgAN and control groups. Notably, BTG2 mRNA expression was significantly reduced in IgAN samples (*p* < 0.001) and was positively correlated with Tfh cells, activated dendritic cells, and M0 macrophages. In addition, violin plot analysis indicated a relative decrease in naïve B cells, Tfh cells, M0 macrophages, and eosinophils in the IgAN group. These results suggest that BTG2, Tfh cells, and M0 macrophages may play critical roles in the pathogenesis of IgAN. In this study, the test set consisted of renal tissue samples, whereas the validation datasets included GSE93798 (glomerular specimens) and GSE35487 (tubulointerstitial specimens). The causal relationship between BTG2 and Tfh/macrophage infiltration was further validated in a Gd‐IgA1‐induced IgAN mouse model, which showed that BTG2 overexpression promoted Tfh cell expansion. In line with previous studies, Tfh cells facilitate IgA production by secreting IL‐21.^65^ BTG2 may enhance IL‐21/STAT3 signaling by antagonizing miR‐25‐3p, which targets STAT3.^66^ Furthermore, BTG2 was found to induce macrophage polarization toward the M2 phenotype. Prior studies have shown that M2 macrophages suppress inflammation by secreting IL‐10,^67^ which may account for the observed reduction in renal inflammation. Collectively, these findings suggest that BTG2 modulates IgAN pathology through dual immunomodulatory mechanisms—namely, Tfh cell expansion and M2 macrophage polarization. Validation analyses also confirmed the association of BTG2 with all types of renal tissue.

In summary, our findings suggest that BTG2 may serve as a potential diagnostic biomarker for IgAN. The positive association of BTG2 expression with Tfh cells and macrophages further supports its possible role in immune regulation and renal protection in this disease. However, these observations still require additional experimental confirmation. A major limitation of the present study is the lack of direct functional immune assays. For example, we did not examine Tfh cell function by blocking the IL‐21/STAT3 pathway under conditions of BTG2 overexpression nor did we use macrophage‐conditioned medium or co‐culture systems to investigate possible paracrine mechanisms. Therefore, it remains unclear whether BTG2 acts directly on specific immune cell subsets or indirectly through intermediate cells or signaling molecules. Future studies should address these issues by establishing immune cell‐specific BTG2 conditional knockout or overexpression mouse models, such as CD4^+^ T cell‐ or macrophage‐specific models, combined with IgAN induction to define the cell‐specific functions of BTG2. In vitro co‐culture systems, including BTG2‐modified renal tubular epithelial cells with naïve T cells or monocytes, together with pathway agonists or inhibitors, should be employed to clarify the mechanisms by which BTG2 regulates Tfh differentiation and macrophage polarization. These approaches may help determine whether the IL‐21/STAT3 axis and TGF‐β/IL‐10 signaling are involved in BTG2‐mediated immune regulation. In addition, future investigations should validate BTG2 expression in both renal tissue and blood samples to further assess its potential clinical utility as a diagnostic biomarker.

## 5 CONCLUSION

In this study, four GEO expression datasets were integrated, and BTG2 was systematically identified and validated as a key diagnostic marker for IgAN through differential expression analysis, functional enrichment, and multiple machine learning approaches. BTG2 expression was significantly reduced in the renal tissues of patients with IgAN and consistently showed strong diagnostic performance across independent datasets, with AUC values greater than 0.94. Its diagnostic value also appeared to be superior to that of other candidate genes, including SOCS3. Further immune infiltration analysis demonstrated that BTG2 expression was positively correlated with Tfh cells and M0 macrophages, suggesting that BTG2 may participate in shaping the immune microenvironment of IgAN. Mechanistically, BTG2 may contribute to disease attenuation by promoting Tfh cell expansion, facilitating macrophage polarization toward an anti‐inflammatory phenotype, and suppressing inflammatory responses. This study systematically highlights the dual relevance of BTG2 in both the diagnosis and immune regulation of IgAN, providing a promising molecular basis for early detection and potential therapeutic intervention.

## AUTHOR CONTRIBUTIONS

Ruimin Ren conceived and designed the study. Min Kou, Mo Dan, Juan Cheng, and Sheng Ge performed the experiments. Sheng Ge, Juan Cheng, and Mo Dan analyzed the data. Min Kou and Mo Dan wrote the manuscript. All authors reviewed and approved the final version of the manuscript.

## CONFLICT OF INTEREST STATEMENT

The authors declare no conflicts of interest.

## ETHICS STATEMENT

All animal procedures were approved by the Institutional Animal Care and Use Committee of Third Hospital of Shanxi Medical University.

## CONSENT FOR PUBLICATION

None.

## Supporting information

Supporting Information S1

## Data Availability

All data generated or analyzed during this study are included in this article and/or its supplementary material files. Further inquiries can be directed to the corresponding author.
