# Supplementary material for: Diagnostic potential of B‐cell translocation gene 2 in IgA nephropathy: Insights from molecular mechanisms
Source: J Cell Commun Signal. 2026 Jul 20;20(3):e70082. doi: 10.1002/ccs3.70082 (PMC13384246; doi:10.1002/ccs3.70082)
Supplement: Supplementary file 1 — Supporting Information S1 [file CCS3-20-e70082-s001.docx]

**
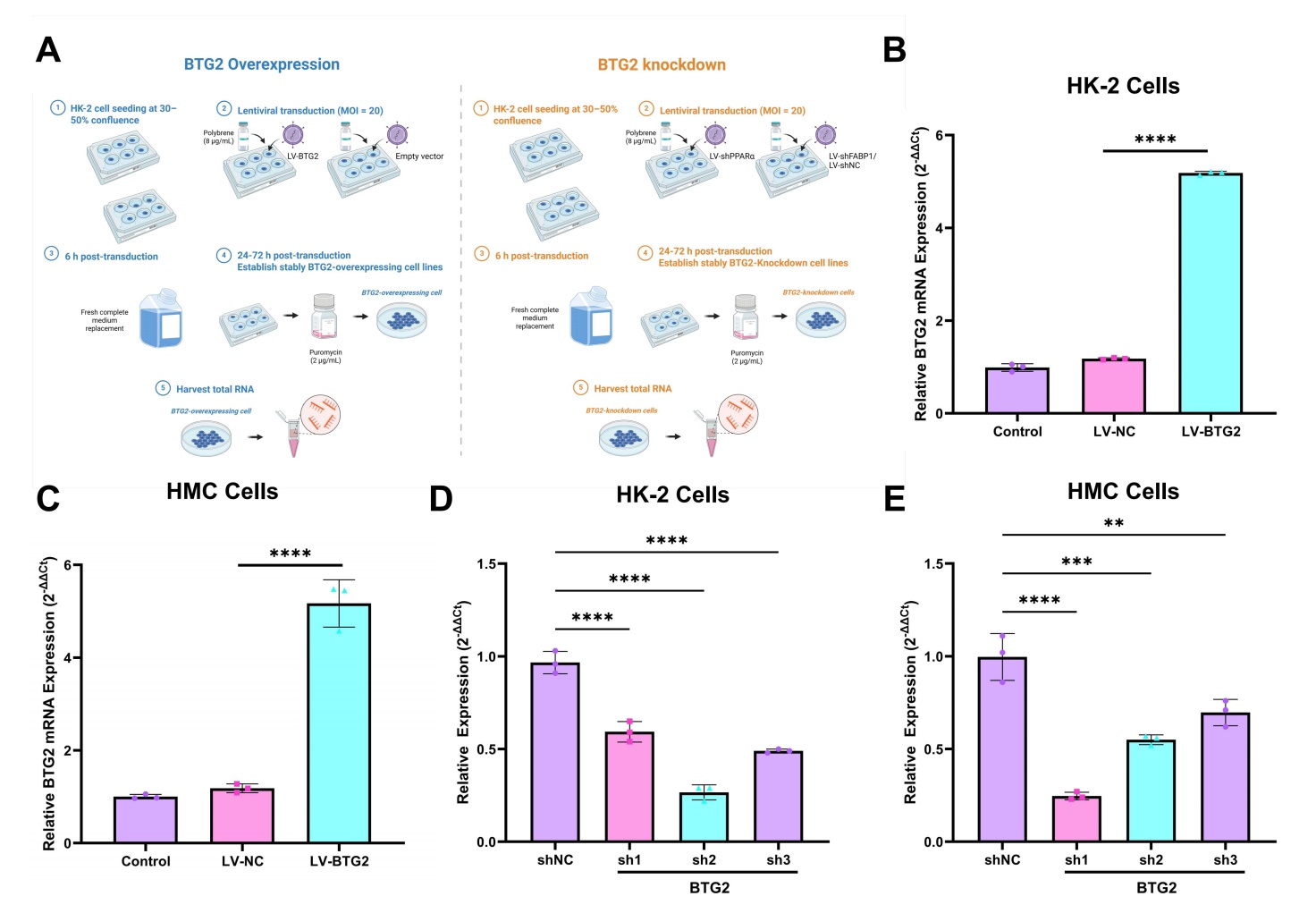
**

**Figure S1. Construction of stable BTG2-overexpressing and BTG2-knockdown cell lines and validation by qPCR.**

**Note: (**A) Schematic diagram of the cell processing workflow for BTG2 overexpression and knockdown. Lentiviruses carrying **BTG2** or **shBTG2** were used to infect HK-2 and HMC cells. After infection, cells were selected with 2 μg/mL puromycin (72 hours for HK-2 and 5 days for HMC) to obtain stable cell lines for subsequent experiments. (B, C) RT-qPCR analysis of **BTG2** expression in HK-2 (B) and HMC (C) cells infected with BTG2-overexpressing lentivirus (LV-BTG2), n = 3. (D, E) Three shRNA sequences targeting **BTG2** were screened and transfected into HK-2 (D) and HMC (E) cells, respectively. RT-qPCR evaluated knockdown efficiency, and the most effective sequence was selected for subsequent experiments, n = 3. Error bars represent SD. ***p <* 0.01, ****p <* 0.001.

**
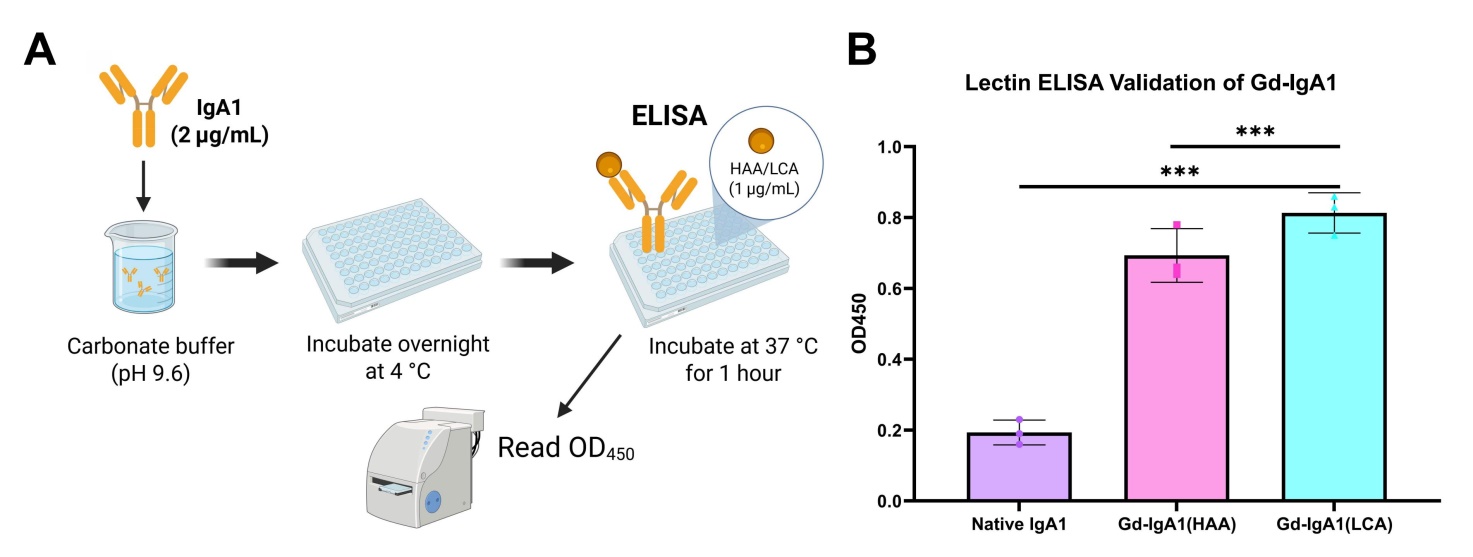
**

**Figure S2. Validation of Gd-IgA1 deglycosylation using lectin-based ELISA.**

**Note: (**A) Schematic illustration of human serum IgA1 enzymatically deglycosylated with β-1,3-galactosidase and neuraminidase to generate Gd-IgA1, followed by detection using HRP-conjugated HAA or LCA lectins in an ELISA assay. (B) ELISA analysis comparing untreated IgA1, Gd-IgA1 detected with HAA, and Gd-IgA1 detected with LCA. All groups were independently repeated three times (n = 3). Data are presented as mean ± SD. ***p <* 0.01, ****p <* 0.001.

**
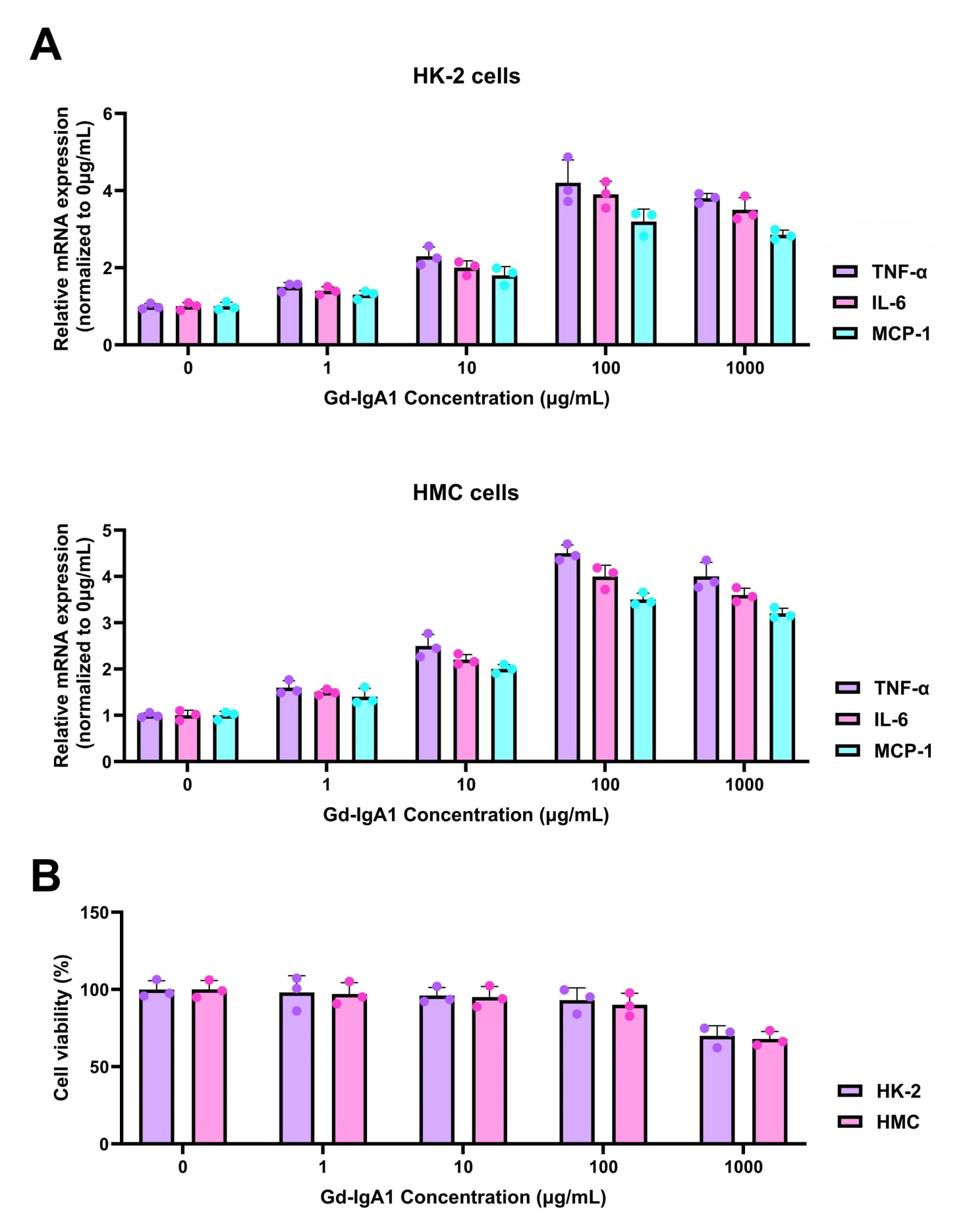
**

**Figure S3. Inflammatory cytokine expression and cytotoxicity evaluation in Gd-IgA1-induced *in vitro* IgAN model.**

**Note: (**A) mRNA expression levels of **TNF-α**, **IL-6**, and **MCP-1** in HK-2 and HMC cells after 24-hour stimulation with varying concentrations of Gd-IgA1 (0, 1, 10, 100, and 1000 μg/mL). Expression levels were normalized to the 0 μg/mL group. Data are shown as mean ± SD, n = 3. (B) Cell viability of HK-2 and HMC cells after 24-hour exposure to the indicated Gd-IgA1 concentrations was assessed using the CCK8 assay. Results are expressed as a percentage relative to the control group. Data are presented as mean ± SD, n = 3.

**
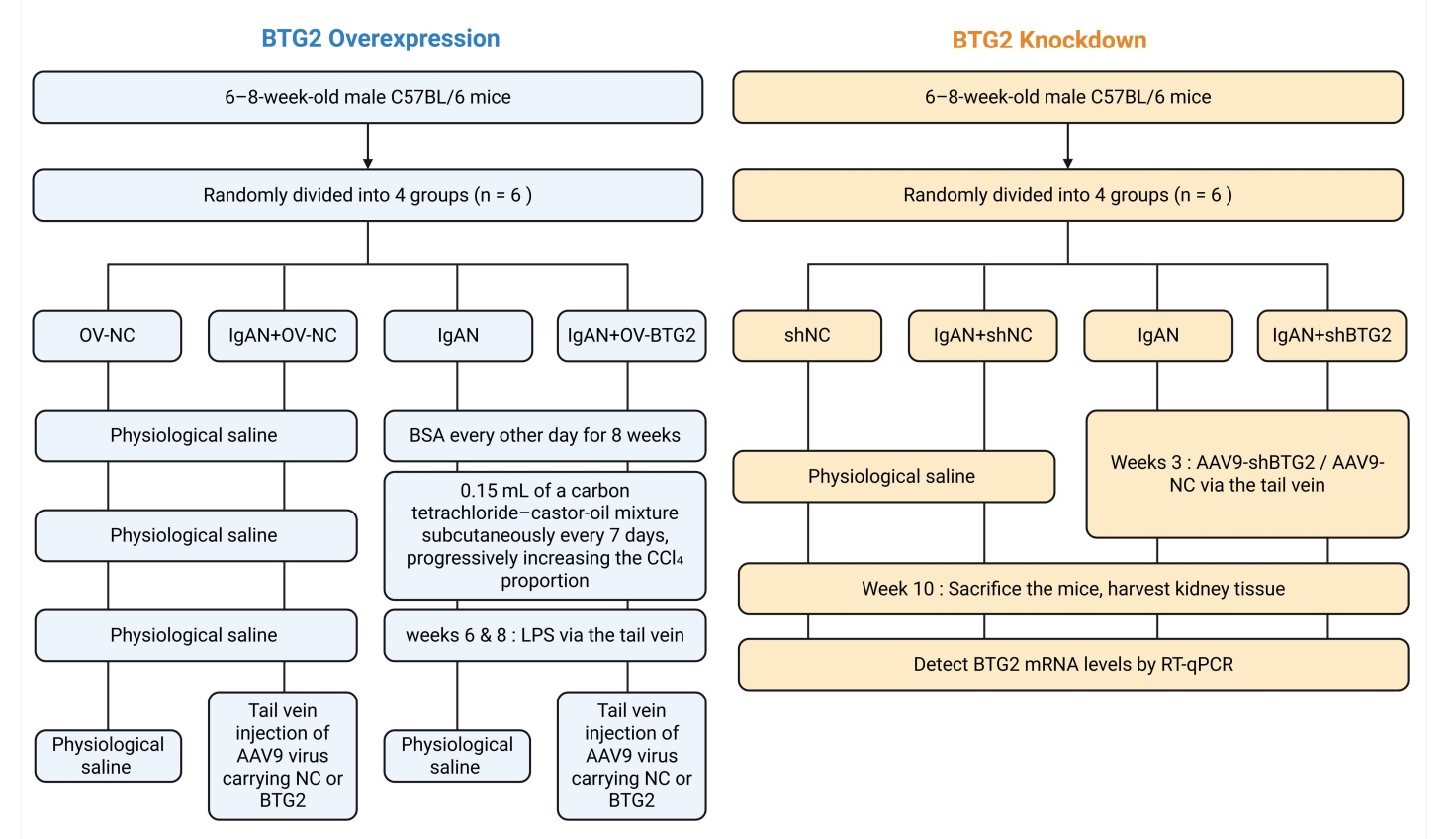
**

**Figure S4. Schematic diagram of animal experiments and IgAN induction protocol.**

**
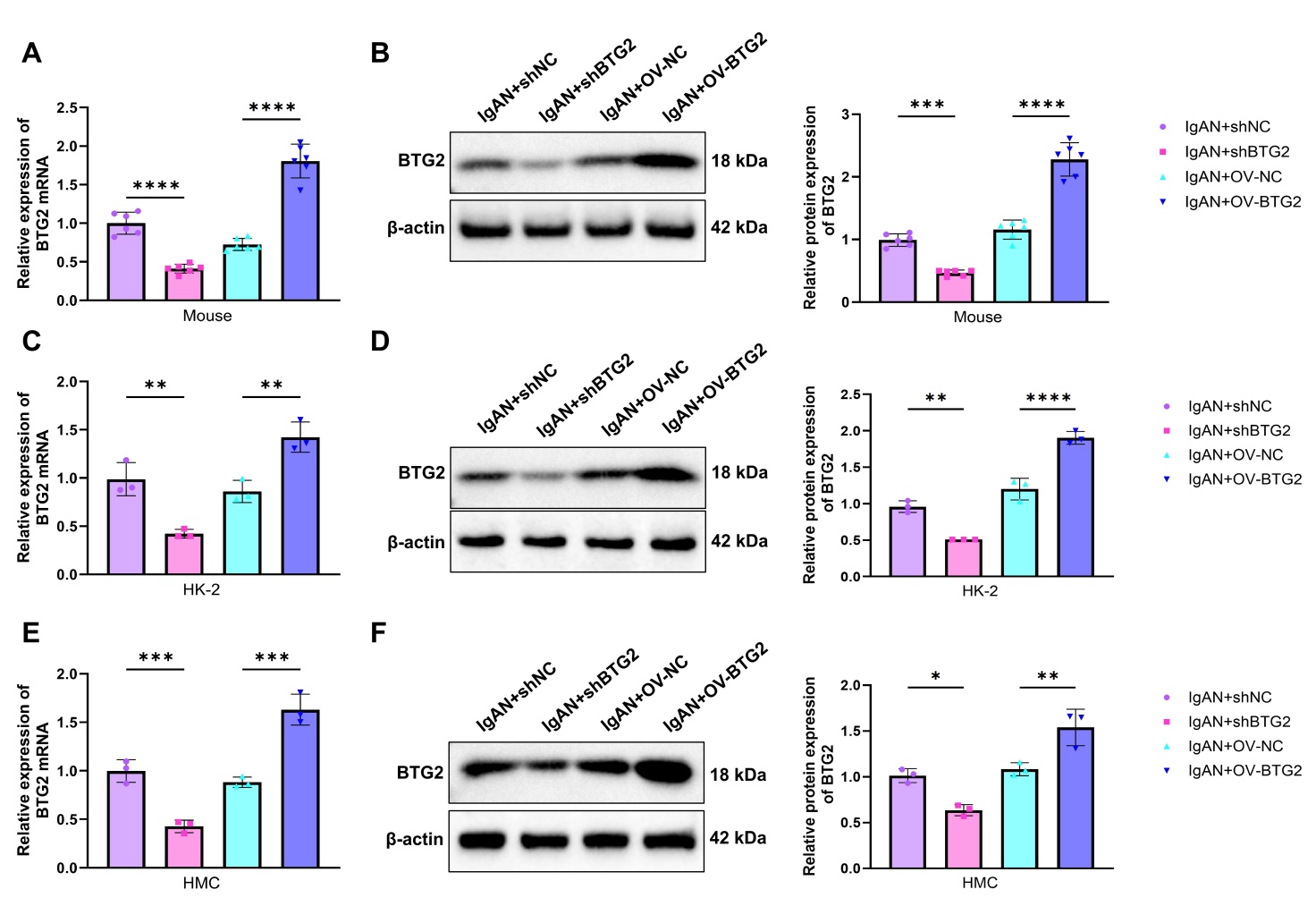
**

**Figure S5. Validation of BTG2 silencing and overexpression efficiency.
Note: (A–B) Western blot and RT-qPCR analysis of BTG2 protein and mRNA expression in mouse glomerular tissues; n = 6 per group. (C–D) Western blot and RT-qPCR analysis of BTG2 protein and mRNA expression in HK-2 cells; n = 3. (E–F) Western blot and RT-qPCR analysis of BTG2 protein and mRNA expression in HMC cells; n = 3. Statistical analysis was performed using one-way ANOVA followed by Tukey's post hoc test. ***p* < 0.01 and ****p* < 0.001.**

**Table S1. Information on Antibodies Used for Flow Cytometry.**

| **Marker** | **Population** | **Recommended Antibody and Fluorochrome** | **Catalog No.** | **Supplier** | **Recommended Dilution** |
| --- | --- | --- | --- | --- | --- |
| CD4 | Tfh parent population | anti-CD4-APC | 100412 | BioLegend | 1:100 |
| CXCR5 | Tfh confirmation | anti-CXCR5-PE (clone L138D7) | 145504 | BioLegend | 1:200 |
| F4/80 | Macrophage parent population | Anti‑F4/80-PerCP‑Cy5.5 | 12‑4801‑82 | eBioscience | 1:100 |
| CD86 | M1 subset | anti-CD86-APC (clone GL-1) | 105012 | BioLegend | 0.25 µg/10⁶ cells |
| CD206 | M2 subset | anti-CD206-BV605 | 141718 | BioLegend | 1:100 |

**Table S2. Primer Sequences for qPCR Analysis.**

| **Gene Name** | **Primer Direction** | **Sequence (5'-3')** |
| --- | --- | --- |
| BTG2（human） | Forward | CACCTGCAAGAACCAAGTGC |
|  | Reverse | CTTGGCTAGGTCTACCCCCT |
| BTG2（mouse） | Forward | CCCTTCGTGTTTCGTGGACT |
|  | Reverse | AGGAGAGGGGATGCAATGGA |
| PPARα（human） | Forward | GAGAAGCTGTCACCACAGTAGCTT |
|  | Reverse | CGCCTCCTTGTTCTGGATGC |
| FABP1（human） | Forward | GCTGGGTCCAAAGTGATCCA |
|  | Reverse | TATGTCGCCGTTGAGTTCGG |
| IL-6（human） | Forward | ACAAGCGCCTTCGGTCC |
|  | Reverse | CATTTGCCGAAGAGCCCTCA |
| IL-6（mouse） | Forward | ACTTCACAAGTCGGAGGCTT |
|  | Reverse | TGACTCCAGCTTATCTCTTGGTTG |
| MCP-1(CCL2)（human） | Forward | CTCTCGCCTCCAGCATGAAA |
|  | Reverse | GGTGTCTGGGGAAAGCTAGG |
| MCP-1 (CCL2)（mouse） | Forward | GTCCCTGTCATGCTTCTGGG |
|  | Reverse | GGACCCATTCCTTCTTGGGG |
| β-Actin（human） | Forward | CCACCATGTACCCTGGCATT |
|  | Reverse | GTCCTCGGCCACATTGTGAA |
| β-Actin（mouse） | Forward | CCCACTCCTAAGAGGAGGATG |
|  | Reverse | CAGACCTGGGCCATTCAGAAA |
| TNF-α（human） | Forward | GACAAGCCTGTAGCCCATGT |
|  | Reverse | GGAGGTTGACCTTGGTCTGG |
| TNF-α（mouse） | Forward | AGGGGATTATGGCTCAGGGT |
|  | Reverse | AGCTCAGCTCCGTTTTCACA |
